# Trends and Hot Spots in Research Related to Rivaroxaban: Bibliometric Analysis

**DOI:** 10.3390/clinpract15100190

**Published:** 2025-10-21

**Authors:** Kornel Pawlak, Łukasz Kruszyna, Anna Wesołowska, Marta Karaźniewicz-Łada

**Affiliations:** 1 Department of Physical Pharmacy & Pharmacokinetics, Poznan University of Medical Sciences, Rokietnicka Street 3, 60-806 Poznan, Poland; 2 Doctoral School, Poznan University of Medical Sciences, Bukowska Street 70, 60-812 Poznan, Poland; 3 Department of Vascular & Endovascular Surgery, Angiology and Phlebology, Poznan University of Medical Sciences, Dluga St. 1/2, 61-848 Poznan, Poland; 4 Department of Pharmacology, Poznan University of Medical Sciences, Rokietnicka Street 3, 60-806 Poznan, Poland

**Keywords:** rivaroxaban, co-citation analysis, co-occurrence analysis, keywords analysis, bibliometric analysis, trends analysis

## Abstract

Background: The number of publications related to rivaroxaban is growing, making it difficult for scientists to review relevant materials. Objectives: This bibliometric analysis is focused on highlighting hot spots and new trends associated with rivaroxaban studies and provides references and guidance for further research. Methods: A comparison between countries, journals, authors, and organizations was performed. Microsoft Excel 2021 and VOSviewer were used to process and visualize data extracted from Web of Science. The time range was set from 1991 to late 2024. A total of 6979 articles were analyzed and bibliometric maps of co-citations of references and co-occurrences of the keywords were built. Results: Relative research interest increased until 2021, when it started to drop. The new trends in publications related to rivaroxaban are associated with a comparison of NOAC therapy outcomes with previously used vitamin K antagonists (warfarin). The research was focused also on new NOAC representatives, medical conditions treated with NOAC, and safety of the therapy. New trending topics are related to ABCB1, peripheral artery disease, direct-acting oral anticoagulants, PCI, and SARS-CoV-2. Conclusions: This bibliometric analysis showed that increasing attention is being paid to the medical conditions treated with NOACs and issues related to the safety of this therapy.

## 1. Introduction

Rivaroxaban, a member of the novel oral anticoagulants (NOACs), offers several advantages over vitamin K antagonists (VKAs), including higher safety and efficiency [1,2,3,4,5]. It is prescribed for patients with nonvalvular atrial fibrillation to prevent venous thromboembolism, stroke, and systemic embolism, as well as for the treatment of deep vein thrombosis (DVT) and pulmonary embolism [4,6]. Regardless of its higher safety profile, rivaroxaban treatment is still associated with multiple adverse effects [7,8,9]. There are also reports describing cases of recurrent thromboses in patients treated with rivaroxaban [10,11]. Moreover, a better bioavailability (39% higher mean AUC) was observed after administration of 15 mg and 20 mg of rivaroxaban with food compared to fasting conditions, but this effect was not confirmed for lower doses (2.5 and 10 mg) [12].

Rivaroxaban is mainly metabolized by CYP3A4 and CYP2J2 enzymes. Hence, the effectiveness of rivaroxaban treatment might be impacted by inhibitors (e.g., verapamil, ketoconazole, diltiazem, clarithromycin) or inducers (e.g., phenobarbital, phenytoin) of the CYP enzymes [13,14,15,16]. Additionally, the influence of BCRP and ABCB1 gene polymorphisms may also affect the response to rivaroxaban treatment [14,17].

An abundant number of factors influencing the therapy’s efficacy encourage further research on this matter. Hence, therapeutic drug monitoring was suggested [18,19,20,21].

Anti-Xa assays are considered suitable for the determination of rivaroxaban. However, these methods measure inhibition of factor Xa, which is not specific to the NOAC [22]. Currently, the HPLC-MS/MS method is a gold standard for measuring rivaroxaban plasma concentrations due to its high specificity, sensitivity, and reproducibility [23,24,25]. There is no strictly established therapeutic window for rivaroxaban concentrations. In the study of Grześk [25], minimal and maximal therapeutic concentrations of rivaroxaban were suggested as 40 and 400 ng/mL, respectively.

A wide range of topics related to rivaroxaban make this subject still actively studied. Moreover, the ambiguous results on factors affecting rivaroxaban effectiveness require further investigation. We believe it is crucial to estimate the direction in which the research areas are developed for prediction of new trends and solutions related to rivaroxaban therapy. Moreover, the previous patent for XARELTO^®^ expired in 2024, which led to new generic medications containing rivaroxaban launching on the market. The increasing availability of rivaroxaban may result in an increased number of studies on its safety and therapeutic effects.

Bibliometric analysis is an excellent method for the scrutiny of publications associated with certain topics. Compared to a literature review, bibliometric analysis provides more objective data based on both quantitative and qualitative results [26,27,28]. This method can help to pinpoint new developing areas and trends regarding a particular subject [26,28]. It is also suitable for analyzing large sets of data with a broad scope [26,28]. These features differentiate it from a literature review, which might be biased and focused on qualitative analysis of small datasets [28].

VOSviewer was developed by van Eck and Waltman at Leiden University [29]. It is a free software used for visualizing bibliometric networks. The networks created can include relations between publications, references, authors, or journals. VOSviewer is also very useful in building clusters, which help in distinguishing particular groups of related data in networks and identify their themes [28,29,30]. It enables researchers to perform both quantitative and qualitative analysis, at the same time revealing the intellectual structure of the data. Built bibliometric networks can help in identifying new trends, cooperations, and connections between studies related to a specific topic which are not visible in more traditional reviews. Additionally, they can be visualized as a density map, if needed. The software can be used to analyze large sets of data, reaching thousands of items, throughout a long timespan. It enables the analysis of data extracted from different sources, such as Web of Science (WOS), SCOPUS, or PubMed databases. Processed data can be extracted as CSV files for further analysis (e.g., in Microsoft Excel).

Rivaroxaban and other drugs included in the NOAC group were widely studied throughout the years. However, we found that the bibliometric analysis is still scarce regarding this topic [31,32,33,34]. Moreover, the bibliometric analyses we found were focused mainly on certain medical conditions in which NOACs are used, but not on the medicines themselves. We found one bibliometric analysis regarding the NOAC group. However, it highlighted research trends in anticoagulation therapy over the last 25 years [31]. Based on citation count, the authors included only 100 publications. It is relatively small number compared to almost 7000 publications included in this paper. Mian et al. [31] analyzed data about the whole NOAC group, while we focused on rivaroxaban. Moreover, the research was performed in April 2019, and it covered data between 1994 and 2019. Considering the rapid increase in research in recent decades, the state of knowledge might change over these few years, and it should be updated. The study did not cover also the research conducted during the COVID-19 pandemic, which might greatly influence the trends and impact of publications related to rivaroxaban. However, an undisputable advantage of this research was the very careful screening through selected publications, ensuring their great impact and association with the subject studied [31].

The main goal of this research is to indicate new trending topics related to rivaroxaban and the most associated journals and organizations. The aim was also to identify the most prominent authors and institutions—leaders in discoveries about the topic, which might be helpful in searching for materials and references in subsequent studies.

## 2. Materials and Methods

### 2.1. Methodology

We used the WOS database to obtain bibliographic data about rivaroxaban on 5 September 2024. Data obtained from WOS was raw data, designed for further bibliometric analysis. The data obtained was processed so that it could be utilized for co-citation and co-occurrence of keyword analysis.

In this study, the WOS database was searched for the “rivaroxaban” phrase, within Core Collection (all editions). The year range covered was 2006–2024 (to the present day). The document type was set as Article. We refrained our research to English articles only.

We used “TS = Topic” tag field for searching through the database. The following query was used:

TS = (“rivaroxaban”).

We chose the following options to refine our results:

rivaroxaban (Topic) and Article (Document Types) and English (Languages).

### 2.2. Data Extraction Process

Data was extracted from the WOS database as separate CSV files and then processed using Microsoft Excel 2021 software. For potential duplicates, we filtered results by doi in Excel, but no duplicates were found. Any record with missing data was removed from the analysis. We exported the search results including the author, title, source, abstract, keywords, addresses, cited references and use and funding, and others. We extracted that information, including all sub-options available. Separately, we extracted data regarding number, year, country, organization, author, journal, meso- and micro-topics, research areas, and publication type. The additional data was extracted for top 10 most productive journals, top 10 most productive organizations, and top 10 most productive countries/territories, including total citations and h-index.

We calculated yearly relative research interest (RRI) using the following formula [35]:

RRI = (number of rivaroxaban publications per year/number of all publications per year).

We obtained the number of all publications per year, using query in Advanced Search Query Builder:

PY = (2006–2024).

### 2.3. Visualization and Map Building

We used VOSviewer version 1.6.20 [29], for the analysis of co-citation of references, co-authorship and co-occurrence of keywords. The data was then extracted as csv files and consecutively analyzed using Microsoft Excel 2021 software.

VOSviewer is freely accessible online, without the necessity of licensing or subscription. The software builds a bibliographic network comprising nodes based on distance. The nodes are grouped into clusters based on similarities between them. We used the software for map building of co-citation of references and co-occurrence of keywords.

Co-citations of references are one of the most crucial methods of bibliographic analysis. It highlights multiple references cited by the same publication [27,28]. We set a threshold for the minimum number of citations per record as 25 to increase the accuracy and transparency of the co-citation map. Out of 84,142, only 972 met the aforementioned criteria.

For visualization of the co-occurrence of the keywords, we used an additional density distribution map as it shows the focus of the research interest. The inclusion criterion was that the keyword had been mentioned at least 5 times. Out of 10,363 keywords, 1422 met the criterion.

## 3. Results

**Figure 1:**
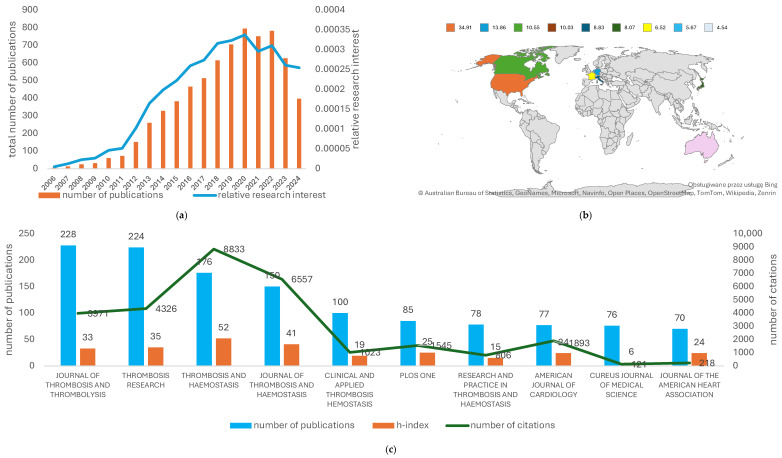
Characteristics of publications related to rivaroxaban: (**a**) RRI and number of publications per year; (**b**) 10 most productive countries, with highest percentage of published papers; (**c**) 10 most productive journals based on number of publications, h-index and number of citations.

As previously noted, rivaroxaban therapy still provides a wide field for further research. Throughout the years, the number of publications in the Web of Science (WOS) related to rivaroxaban increased, reaching its peak in 2020 (Figure 1). Therefore, we believe it is important to identify new trends and directions in which scientific research on rivaroxaban is heading. We performed bibliometric analysis with Microsoft Excel regarding the overall number of publications, the most productive sources of publications (countries, journals, organizations, authors, funders), and the research areas. Then, we conducted analysis of co-citations of references and co-occurrence of keywords, using VOSviewer software (version 1.6.20). We believe it is the first such analysis about rivaroxaban which uses both VOSviewer and WOS database. Our study provides a general overview on studies associated with rivaroxaban.

### 3.1. Overall Number of Publications Related to Rivaroxaban

When we searched for “rivaroxaban”, we obtained a total of 12,445 results from the WOS Core Collection. After filtering for Article Document type, the number decreased to 7525 results. Finally, we refined the records to English only, obtaining 6979 results. Then, we proceeded with further analysis. The total number of citations of publications related to rivaroxaban was 191,680 (141,442 without self-citations). The h-index equaled 161, and the average citation per article was 27.47 times.

### 3.2. Number of Publications per Year and RRI

The first publications related to rivaroxaban appeared in 2006. The most productive years were 2020 (795 publications), 2023 (785 publications), and 2021 (751 publications). The number of publications per year as well as RRI are displayed in Figure 1a.

### 3.3. Countries of Publications

The United States of America (USA) published the greatest number of papers (2436, 34.90%). The next was Germany (967, 13.86%), followed by Canada (736, 10.55%), and England (736, 10.55%). The highest number of citations obtained was by USA (110,246), followed by Germany (65,576) and Canada (60,376. USA, Germany, and Canada had the highest h-index, equaling 133, 111, and 98, respectively. Figure 1b and Table S1 depict the 10 most productive countries, with their total number of publications, total number of citations, and h-index.

### 3.4. Most Productive Journals

The most productive journal was the Journal of Thrombosis and Thrombolysis, which published 228 (3.27%) papers. Thrombosis Research and Thrombosis and Haemostasis were next behind, with 224 (3.21%) and 176 (2.52%) papers, respectively. Figure 1c shows the 10 most productive journals, including their h-index and number of citations.

### 3.5. Most Productive Organizations

Harvard University produced the most papers about rivaroxaban (412, 5.90%), followed by McMaster University (404, 5.79%) and Bayer Ag (402, 5.76%). The number of citations corresponded with publication number, as the most citations obtained were by Harvard University (43,197), followed by McMaster University (40,201) and Bayer Ag (38,888).

**Figure 2:**
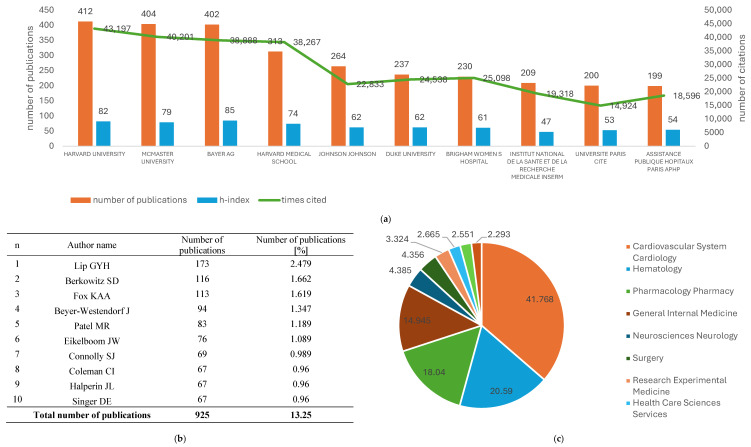
Summary of (**a**) 10 most productive organizations based on number of publications, h-index and number of citations; (**b**) most productive authors based on number of publications; (**c**) most frequently assigned research areas.

Even though Harvard University had a higher number of citations and published papers, it had a lower h-index (h-index = 82), than Bayer Ag (h-index = 85). Figure 2a shows the top 10 most productive organizations, including the h-index and total number of citations.

### 3.6. Most Productive Authors

Our study revealed that 36,696 authors contributed to the research about rivaroxaban. The summed-up research of 10 top authors equals 13.25% of all publications about rivaroxaban. The most papers were published by Lip GYH (173, 2.48%), followed by Berkowitz SD (116, 1.66%) and Fox KAA (113, 1.62%). The full list of the 10 most productive authors is displayed in Figure 2b.

### 3.7. Most Prominent Funders

The highest amount of funding for papers related to rivaroxaban was provided by Bayer Ag (650, 9.31%), then Pfizer (376, 5.39%), and Bristol Myers Squibb (355, 5.09%). The list of the 10 most prominent funders is available in the Supplementary Materials (Table S2).

### 3.8. Subjects of Area, Meso-Topics, and Micro-Topics Analysis

According to WOS research areas, most papers were related to Cardiovascular System/Cardiology (2915, 41.77%), Hematology (1437, 20.59%), and Pharmacology/Pharmacy (1259, 18.04%). Figure 2c illustrates the distribution of the 10 most frequently assigned categories by WOS.

The most frequent meso-topics were Cardiac Arrhythmia (3949, 56.58%), Blood Clotting (1592, 22.81%), and Cardiology Circulation (170, 2.44%). The full list of the 10 most frequently assigned meso-topics is available in Table 1.

Regarding Micro-topics, the highest record count belonged to Atrial Fibrillation (3916 mentions, 56.11%), Pulmonary Embolism (1098, 15.73%), and Antiphospholipid Syndrome (237, 3.40%). The full list of the 10 most frequently assigned micro-topics is available in Table 2.

### 3.9. Co-Citations References

**Figure 3:**
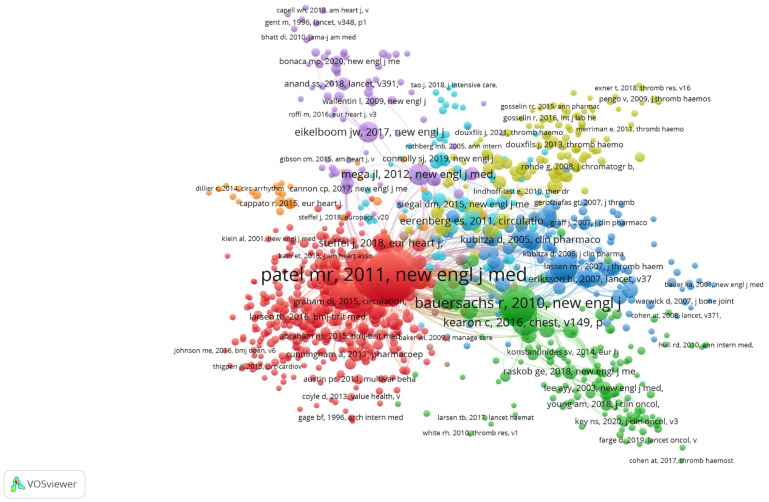
Network map of co-cited references in rivaroxaban studies.

We distinguished 7 clusters (Figure 3). The first cluster comprised 258 items. It focused on the comparison of NOAC therapy efficacy, with vitamin K antagonists (e.g., warfarin). It was dedicated also to new guidelines regarding therapy with NOAC.

The second cluster had 226 co-cited references and focused mainly on the clinical characteristics of NOAC (including rivaroxaban), assays used for determination of the therapy’s efficacy, and NOAC pharmacokinetics.

The third cluster comprised 161 items and was associated with NOAC therapy directed at venous thromboembolism and pulmonary embolism.

The fourth cluster included 129 publications and was primarily dedicated to atrial fibrillation.

The fifth cluster comprised 93 publications. It was devoted to the prevention of thromboembolism and the safety of the therapy with NOAC. It was also centered around thromboprophylaxis and the comparison of NOAC to other medicines (e.g., enoxaparin).

The sixth cluster consisted of 75 items, and it was focused on the co-administration of rivaroxaban with antiplatelet agents in dual antithrombotic therapy (e.g., clopidogrel, aspirin).

The seventh cluster consisted of 30 publications. The publications gathered in this cluster centered around NOAC therapy in patients after cardioversion and atrial fibrillation.

### 3.10. Co-Occurrence of Keywords

**Figure 4:**
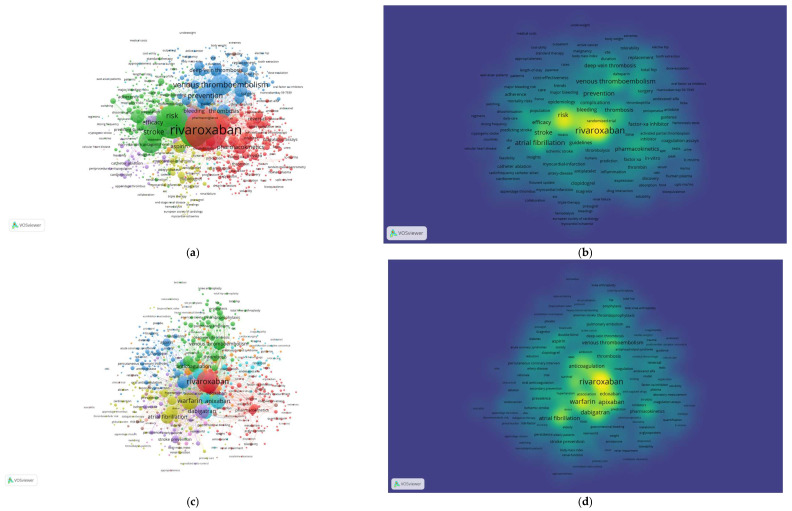
Network map of co-occurrence of keywords for overall analysis from 2006 to 2024 (**a**) and density distribution (**b**). Network map of co-occurrence keywords, between 2021 and 2024 (**c**) and density distribution (**d**).

We used the co-occurrence of keywords to analyze the different directions in which the research is conducted. First, we divided the 2006–2024-time range into four smaller time periods (2006–2010, 2011–2015, 2016–2020, and 2021–2024). We analyzed these sections separately and altogether (Figure 4 and Figure S1). The data was extracted from VOSviewer and analyzed by Microsoft Excel 2021. For overall analysis, out of 10,363 keywords, only 1422 met the criteria (keyword appeared in at least five publications).

The keywords analysis was performed using VOSviewer. The study was performed for all the years and separately for specific time periods (2006–2010, 2011–2015, 2016–2020 and 2021–2024). Regarding overall analysis, the most frequently appearing keyword was “rivaroxaban”, which occurred in papers 4524 times. It was followed by warfarin (2738), dabigatran (2171), apixaban (1796), and atrial fibrillation (1426), making the list of the five most frequent keywords in papers, related to rivaroxaban.

The most frequent keywords in 2021–2024 are as follows: “rivaroxaban” (1624 publications), “warfarin” (933 publications), “apixaban” (650 publications), “dabigatran” (599 publications), and “atrial fibrillation” (495 publications).

The most common keywords, in the last 4 years and related to other drugs, are as follows: “warfarin” (933), “dabigatran” (599), and “apixaban” (650). The new keywords that appeared in the 2021–2024 time range were “ABCB1” (13 times), “peripheral artery-disease” (12 times), “direct-acting oral anticoagulant” (7 times), “PCI” (10 times), and “SARS-CoV-2” (10 times). Regarding medical conditions, the most frequently appearing keywords are as follows: “atrial fibrillation” (495) and “atrial-fibrillation” (299), “stroke” (379), “venous thromboembolism” (356), “thrombosis” (194), “thromboembolism” (115), “deep-vein thrombosis” (85), and “hemorrhage” (80). Table 3 shows a summary of 10 hotspot keywords in publications divided by time periods. Tables S3 and S4 show 25 most frequently used keywords for each time period and for overall analysis.

## 4. Discussion

This bibliometric analysis aimed to identify the main research areas and emerging trends related to publications on rivaroxaban, providing a comprehensive overview and guidance for future studies. The obtained results form the basis for an in-depth discussion of the potential factors shaping these trends and their significance for clinical practice.

### 4.1. New Trends in Research Related to Rivaroxaban

The number of publications increased from 2006 to 2019. Then, it stayed on approximately the same level until 2021, when it started to decrease (Figure 1). RRI mirrored this observation, as it increased to reach maximal values in 2019. Then it gradually started to decrease in the last 3 years (Figure 1). These results might be derived from the rapidly increasing interest in the new therapeutic approach presented by launching rivaroxaban on the market in 2008 [36]. Rivaroxaban provided an alternative for commonly used vitamin K antagonists (e.g., warfarin). Then, probably due to launching new representatives of the NOAC group (e.g., apixaban), the RRI started to decrease. Moreover, some studies might suggest that rivaroxaban is not as safe as other NOACs, which might hinder research on rivaroxaban therapy [37,38]. On the other hand, it might also increase the number of studies focusing on the comparison between rivaroxaban and potential alternatives. One of the many reasons for the upholding interest in rivaroxaban therapy between 2019 and 2022 might be the COVID-19 pandemic [39,40], as rivaroxaban might be used for treating pulmonary embolism, which is one of the COVID-19 symptoms [41]. In 2023 and 2024, the RRI was relatively smaller than previously observed. However, the data we used for analysis did not include all publications from 2024, so there is the possibility that this trend might reverse.

The most publications came from the United States of America (34.91%). The USA was followed by Germany (13.86%) and Canada (10.55%). These three countries also had the highest h-index and highest citation count. Even though the People’s Republic of China was fifth in the top ten countries, it has the lowest h-index (44) [42], which might relate to lower quality of published research, compared to other countries. Despite increasing number of researchers and published papers, China still struggles with problems such as research integrity, invented data, or plagiarism [42]. However, the quality of the research conducted in China is likely to improve. More and more emphasis is put on international collaborations, and a dire need for more rigorous evaluation system was identified [42]. Italy was sixth on the list and it had the lowest citation count. However, Italy presented the fourth-highest reported h-index, which indicates the high quality of the published research. Similarly, France, despite having a smaller publication count than Japan and the People’s Republic of China, had a higher h-index (75 compared to 47 and 44, respectively). It might be an indication of high-quality and cutting-edge topics covered by published research.

Regarding articles related to rivaroxaban, the most prominent organization was Harvard University with a record count of 5.90% of publications. However, it was closely followed by McMaster University (5.79%) and Bayer Ag (5.76%). Hence the next crucial publications are expected to originate from these three organizations. Notably, that the presence of Bayer Ag, might be related to it being the discoverer of XARELTO^®^, whose active pharmaceutical ingredient is rivaroxaban.

*Journal of Thrombosis and Thrombolysis* had the highest publication count at 228 (3.27%), followed by Thrombosis Research (224, 3.21%) and Thrombosis and Haemostasis (176, 2.52%). Thrombosis and Haemostasis was third on the list; however, it had the highest citation count (8833) and the highest h-index (52). Although the Journal of Thrombosis and Haemostasis was fourth on the list, it had the second-highest h-index and citation count (41 and 6557, respectively). High citation count and high h-index of Thrombosis and Haemostasis and Journal of Thrombosis and Haemostasis reflect high quality of the articles published in these internationally recognized specialist journals. Even though Cureus Journal of Medical Science published 76 papers, it had the lowest h-index and citation count (6 and 121, respectively). This result was lower than presented by the Journal of The American Heart Association, which was the last on the list, regarding the publication count. However, considering the h-index, it was sixth on the list. What seems to be interesting is that the analysis of co-citations of references analysis revealed that the top three co-cited articles were published in The New England Journal of Medicine (30 publications published, 24,864 times cited, h-index-30), which was not on the list of top 10 most productive journals. However, both the h-index of published papers and their citation count prove the high quality of the research and the journal recognizability.

According to WOS research areas, most papers were related to Cardiovascular System/Cardiology (41.77%), Hematology (20.59%), and Pharmacology/Pharmacy (18.04%). We believe that the future research will also be related to these subjects.

### 4.2. Hot Spots in Research Related to Rivaroxaban

We conducted analysis of the keyword co-occurrences to identify hotspot topics related to rivaroxaban. Considering overall analysis, the most frequently appearing keyword was “rivaroxaban”, which occurred in 4524 publications. It was followed by warfarin (2738), dabigatran (2171), apixaban (1796), and atrial fibrillation (1426), making the list of the five most frequent keywords in papers related to rivaroxaban.

Regarding studied diseases, at first, the most frequently researched disease was “venous thromboembolism” (83 times), being even more frequent than “rivaroxaban” (81 publications). Gradually, over the years, it appeared less, till being replaced after 2016, by “atrial fibrillation” which took the first place (677 publications, compared to 503). Another disease was “deep vein thrombosis”. However, its popularity deteriorated gradually, after 2010, now being the least frequent of the three diseases. It might be related to the fact that primary indication for treatment with rivaroxaban was prevention of deep vein thrombosis and pulmonary embolism. This may be the cause for which at first most publications were centered around these medical conditions. However, with time, new applications for rivaroxaban were sought, resulting in increasing popularity of different medical conditions (e.g., atrial fibrillation). Another keyword related to medical conditions was “COVID-19”, which appeared recently (30 publications in 2016–2020 and 19 in 2021–2024). However, it never reached the same popularity compared to the beforementioned medical conditions. It might be related to the fact that papers about COVID-19 were strictly published around the pandemic.

The most common keywords in the last 4 years and related to other drugs, were the following: “warfarin” (933), “dabigatran” (599), and “apixaban” (650). Compared to previous years, before 2021, “dabigatran” appeared more often (1108), than “apixaban” (812). After 2021, the situation has reversed, making “apixaban” a more frequently appearing keyword. After analysis of the 2006–2010 year range, we found that one of the most common keywords was “enoxaparin” (472). However, after 2010, this keyword was less frequently used (195 in 2011–2015, 136 in 2016–2020 and 86 times in 2021–2024 time periods, respectively). Based on these results, we concluded that new publications will be related mostly to representatives of the NOAC group and medical conditions (e.g., atrial fibrillation).

The entirely new keywords that appeared in the 2021–2024 time range were “ABCB1” (13 times), “peripheral artery-disease” (12 times), “direct-acting oral anticoagulant” (7 times), “PCI” (10 times), and “SARS-CoV-2” (10 times). The appearance of these topics in publications indicates that new research related to rivaroxaban is still being conducted.

Regarding medical conditions, the most frequently appearing keywords are as follows: “atrial fibrillation” (495 publications) and “atrial-fibrillation” (299 publications), “stroke” (379 publications), “venous thromboembolism” (356 publications), “thrombosis” (194 publications), “thromboembolism” (115 publications), “deep-vein thrombosis” (85 publications), and “hemorrhage” (80 publications). New trending topics related to medical conditions are as follows: peripheral artery-disease (12 publications), SARS-CoV-2 (10 publications), “arterial thromboembolism” (8 publications), and “coronary heart disease” (8 publications). New medical conditions, appearing in publications related to rivaroxaban, may lead to conclusions about finding new applications for the NOAC.

We observed that interest in topics related to new analytical methods, such as “LC-MS/MS” first appeared between 2011 and 2015 (14 publications). Then it increased between 2016 and 2020 (25 publications). The number of papers has been decreasing since 2021, with the “UPLC-MS/MS” keywords, appearing in only eight publications in 2021–2024 time range, compared to 11 publications in 2016–2020. We observed also that “performance liquid-chromatography” and “liquid-chromatography” appeared less frequently, when compared 2016–2020 and 2021–2024 time periods (17 to 9 and 8 to 5 publications, for both keywords, respectively). This observation might be derived from the fact that previous publications were related to searching and developing new methods for rivaroxaban measurements [43,44,45]. However, currently, the HPLC-MS/MS method is considered “gold standard”, which might result in fewer publications regarding developing new analytical methods [23,46].

### 4.3. The Analysis of Co-Cited References

The co-citation of reference analysis revealed the three most co-cited articles. In the first cluster, the publication entitled “Rivaroxaban versus warfarin in nonvalvular atrial fibrillation” was published 1973 times in *The New England Journal of Medicine*, by Patel et al. [1]. This publication gained also the third highest number of citations (6300 citations in total and 450 citations per year, Table S5). The research focused on the comparison of rivaroxaban and warfarin therapy. The authors underlined that regardless of high efficacy of warfarin therapy, it requires constant monitoring and dose adjustment. The authors conducted the study which included 14,264 patients suffering from nonvalvular atrial fibrillation. The participants received either 20 mg of rivaroxaban or an adjusted dose of warfarin. The research proved that in patients with atrial fibrillation, rivaroxaban therapy was not inferior to warfarin for the prevention of stroke or systemic embolism. Moreover, they noticed that severe side effects (such as intracranial and fatal bleeding) appeared less frequently in patients treated with rivaroxaban. On one side, these findings provided evidence of the high efficacy of rivaroxaban therapy. On the other side, regardless of the more convenient treatment, there was no significant difference in the risk of major bleeding between patients taking rivaroxaban and those administrated with warfarin.

The second most co-cited publication was “Dabigatran versus warfarin in patients with atrial fibrillation” (1746 times, 8488 times cited, 530 citations per year), published in *The New England Journal of Medicine*, by Connolly et al. (2). The authors wanted to compare warfarin therapy outcomes to those obtained during NOAC treatment. They focused their research on dabigatran. The studied group was 18,113 patients, treated with either adjusted dose of warfarin or 110–150 mg of dabigatran. They found that in patients with atrial fibrillation, treatment with 110 mg of dabigatran was associated with a similar risk of stroke and systemic embolism, compared to warfarin (1.53% to 1.69% risk of stroke or systemic embolism). For 110 mg dose of dabigatran, they also observed lower risk of major bleeding, compared to warfarin (2.71% to 3.36%). The 150 mg dose of dabigatran was related to a lower risk of stroke and systemic embolism, but a similar risk of major hemorrhage (3.11% and 3.36% for dabigatran and warfarin, respectively). The conclusions of this research were crucial for developing new therapeutic approaches and further characterizing NOACs.

The third most co-cited article appeared also in cluster 1 (1691 times co-cited, cited 6690 times with 514 citations per year). Its title was “Apixaban versus warfarin in patients with atrial fibrillation”, and it was published in *The New England Journal of Medicine*, by Granger et al. [3]. As before, authors focused on treatment comparison between warfarin and NOAC. This study highlighted the advantages of apixaban treatment in patients suffering from atrial fibrillation. The studied group consisted of 201 participants treated with 5 mg of apixaban twice a day or adjusted dose of warfarin. The primary outcome was ischemic/hemorrhagic stroke or systemic embolism. The authors found that apixaban was superior to warfarin in primary outcome (1.27% to 1.60% per year). They also found that apixaban treatment was associated with a lower risk of major bleeding, compared to warfarin therapy (2.13% to 3.09% per year). The study delivered another evidence of similar efficacy of NOAC treatment compared to vitamin K antagonist and its better safety profile. However, at the same time, it proved also that this new approach did not eliminate potential side effects. In doing so, it highlighted the still-existing demand for drug monitoring and individualizing the therapy depending on the patient’s condition.

## 5. Conclusions

In this bibliometric analysis, we presented the trend of RRI in publications related to rivaroxaban. We observed that RRI grew till 2020 and then decreased henceforth. The USA was the most productive country, with the highest h-index and citation count. Although People’s Republic of China was fifth in the top 10 countries, its articles may be regarded as of lower quality compared to the other countries on the list. Harvard University, McMaster University and Bayer Ag, had both the highest recognition and number of publications. *Journal Of Thrombosis and Thrombolysis, Thrombosis Research* and *Thrombosis and Haemostasis* were the most productive journals. The new trends in publications related to rivaroxaban are associated with a comparison of NOAC therapy outcomes, with previously used vitamin K antagonists (warfarin). Based on the co-occurrence of the keywords, we observed that research was focused mainly on new NOAC representatives (apixaban, dabigatran), medical conditions treated with NOAC (atrial fibrillation, venous thromboembolism), and safety of the therapy (risk, management, safety). New trending topics are related to ABCB1, peripheral artery disease, direct-acting oral anticoagulant, PCI, and SARS-CoV-2. Other trending topics related to medical conditions are arterial thromboembolism, coronary heart disease, gastrointestinal hemorrhage, and nephrotic syndrome. New trends related to medical procedures are as follows: “electrical cardioversion”, “angiography” and “inferior vena cava filter”. Presented research might help to indicate new trending topics related to rivaroxaban and the most associated journals and organizations. It includes also data about possible, most prominent authors and institutions—leaders in discoveries about the topic, which might be helpful in searching for materials and references in subsequent studies. At the same time, it provides a one-point overview regarding already explored subjects and develops different directions of the studies conducted on rivaroxaban. We believe that the presented results will help identify knowledge gaps as well as develop new ideas for future research.

## 6. Limitations

First of all, the study was conducted in a specific time period, hence the results can change in the future. We did not include all papers published in 2024, hence the results might differ at the end of this year. This is why this research should be updated in the future. Moreover, the publication process is very often very time-consuming; hence, we excluded publications which were not published. However, at the time of writing this article, they might be under consideration or review. Second, for the co-occurrence of keywords, our threshold was set to five publications, which might exclude more recently published papers which did not meet these criteria. The same applies to the co-citation of the references, as the latest, valuable research might not be included. The third limitation was estimating the popularity/performance of the research based on the h-index as it might lead to false conclusions about its quality. Moreover, we based our analysis on one database only (WOS) although there are many others suitable for extracting bibliometric data (e.g., SCOPUS, Dimensions). The abovementioned limitations might result in overrepresentation of certain regions or underrepresentation of emerging topics.

## Figures and Tables

**Table 1:** The list of 10 most frequently assigned meso-topics.

Citation Meso-Topics	Record Count	% of 6979
Cardiac Arrhythmia	3949	56.58
Blood Clotting	1592	22.81
Cardiology Circulation	170	2.44
Vascular, Cardiac, and Thoracic Surgery	132	1.89
General Cardiology	107	1.53
Liver Diseases	59	0.85
Drug Delivery Chemistry	58	0.83
Soft Tissue, Bone, and Nerve Cancers	56	0.80
Virology-General	51	0.73
Pharmacology and Toxicology	39	0.56

**Table 2:** The list of 10 most frequently assigned micro-topics.

Citation Micro-Topics	Record Count	% of 6979
Atrial Fibrillation	3916	56.11
Pulmonary Embolism	1098	15.73
Antiphospholipid Syndrome	237	3.40
Acute Myocardial Infarction	148	2.12
Tissue Factor	115	1.65
Peripheral Arterial Disease	98	1.40
Coronavirus	51	0.73
Heparin-induced Thrombocytopenia	51	0.73
Portal Hypertension	45	0.65
Solid Dispersion	42	0.60

**Table 3:** Ten hotspot keywords in publications in 2006–2010, 2011–2015, 2016–2020, and 2021–2024 time periods.

Time Period	Occurrences	Time Period	Occurrences	Time Period	Occurrences	Time Period	Occurrences
2021–2024		2016–2020		2011–2015		2006–2010	
rivaroxaban	1624	rivaroxaban	2042	rivaroxaban	777	venous thromboembolism	83
warfarin	933	warfarin	1323	warfarin	466	rivaroxaban	81
apixaban	650	dabigatran	1108	dabigatran	442	prevention	66
dabigatran	599	apixaban	812	apixaban	319	enoxaparin	56
atrial fibrillation	495	atrial fibrillation	677	venous thromboembolism	287	deep-vein thrombosis	45
risk	437	management	514	atrial fibrillation	247	double-blind	45
management	387	stroke	507	prevention	211	thromboprophylaxis	43
stroke	379	venous thromboembolism	503	enoxaparin	195	bay-59-7939	40
safety	372	risk	501	atrial-fibrillation	171	dabigatran etexilate	36
venous thromboembolism	356	safety	501	thromboprophylaxis	167	factor-xa inhibitor	34

## Data Availability

Raw data were extracted from WOS. Derived data supporting the findings of this study are available from the corresponding author.

## Supplementary Materials

The following supporting information can be downloaded at https://www.mdpi.com/article/10.3390/clinpract15100190/s1, Table S1. 10 most productive countries, with highest percentage of published papers; Table S2. 10 most prominent funders of the papers, related to rivaroxaban; Table S3. 25 most frequently occurring keywords in publications between 2006–2024; Table S4. 25 most frequently occurring keywords in publications in 2006–2010, 2011–2015, 2016–2020 and 2021–2024 time periods; Table S5. Top 10 most frequently cited articles; Figure S1. Network map of rivaroxaban co-occurrence keywords including density distribution, between 2006–2010 (a), 2011–2015 (b) and 2016–2020 (c).

## Abbreviations

The following abbreviations are used in this manuscript:

NOAC	novel oral anticoagulants
VKA	vitamin K antagonists
DVT	deep vein thrombosis
WOS	Web of Science
