# Supplementary material for: Trends and Hot Spots in Research Related to Rivaroxaban: Bibliometric Analysis
Source: Clin Pract. 2025 Oct 21;15(10):190. doi: 10.3390/clinpract15100190 (PMC12564707; doi:10.3390/clinpract15100190)
Supplement: Supplementary file 1 [file clinpract-15-00190-s001.zip › clinpract-3906641-supplementary.pdf]

Table S1. 10 most productive countries, with highest percentage of published papers

| Countries/Regions | Publications | % of 6979 | h-index | Citation count |
|---|---|---|---|---|
| USA | 2436 | 34.91 | 133 | 110246 |
| GERMANY | 967 | 13.86 | 111 | 65576 |
| CANADA | 736 | 10.55 | 98 | 60376 |
| ENGLAND | 736 | 10.55 | 88 | 51554 |
| PEOPLES R CHINA | 700 | 10.03 | 44 | 11448 |
| ITALY | 616 | 8.83 | 75 | 3941 |
| JAPAN | 563 | 8.07 | 47 | 17569 |
| FRANCE | 455 | 6.52 | 75 | 36527 |
| NETHERLANDS | 396 | 5.67 | 73 | 39494 |
| AUSTRALIA | 317 | 4.54 | 67 | 28811 |

Table S2. 10 most prominent funders of the papers, related to rivaroxaban.

| Funding Agencies | Record Count | % of 6979 |
|---|---|---|
| BAYER AG | 650 | 9.31 |
| PFIZER | 376 | 5.39 |
| BRISTOL MYERS SQUIBB | 355 | 5.09 |
| BOEHRINGER INGELHEIM | 335 | 4.80 |
| DAIICHI SANKYO COMPANY LIMITED | 269 | 3.85 |
| UNITED STATES DEPARTMENT OF HEALTH HUMAN SERVICES | 234 | 3.35 |
| BAYER HEALTHCARE PHARMACEUTICALS | 222 | 3.18 |
| NATIONAL INSTITUTES OF HEALTH NIH USA | 215 | 3.08 |
| JOHNSON JOHNSON | 198 | 2.84 |
| JOHNSON JOHNSON USA | 198 | 2.84 |

Table S3. 25 most frequently occurring keywords in publications between 2006-2024.

| Keywords 2006-2024 | Cluster | Occurrance |
|---|---|---|
| rivaroxaban | 1 | 4524 |
| warfarin | 2 | 2738 |
| dabigatran | 2 | 2171 |
| apixaban | 2 | 1796 |
| atrial fibrillation | 2 | 1426 |
| venous thromboembolism | 3 | 1229 |
| risk | 2 | 1077 |
| management | 5 | 1057 |
| stroke | 2 | 1051 |
| safety | 2 | 1035 |
| prevention | 3 | 962 |
| atrial-fibrillation | 6 | 865 |
| anticoagulants | 1 | 845 |
| anticoagulation | 5 | 831 |
| oral anticoagulants | 2 | 801 |
| direct oral anticoagulants | 1 | 725 |
| therapy | 2 | 720 |
| efficacy | 5 | 574 |
| stroke prevention | 2 | 544 |
| thrombosis | 3 | 517 |
| pharmacokinetics | 1 | 510 |
| metaanalysis | 2 | 502 |

| | | |
|---|---|---|
| edoxaban | 9 | 487 |
| enoxaparin | 3 | 473 |
| thromboprophylaxis | 3 | 457 |

*Table S4. 25 most frequently occurring keywords in publications in 2006-2010, 2011-2015, 2016-2020 and 2021-2024 time periods.*

| Time period 2021-2024 | Cluster | Occurrences | Time period 2016-2020 | Cluster | Occurrences | Time period 2011-2015 | Cluster | Occurrences | Time period 2006-2010 | Cluster | Occurrences |
|---|---|---|---|---|---|---|---|---|---|---|---|
| rivaroxaban | 1 | 1624 | rivaroxaban | 1 | 2042 | rivaroxaban | 1 | 777 | venous thromboembolism | 1 | 83 |
| warfarin | 4 | 933 | warfarin | 4 | 1323 | warfarin | 2 | 466 | rivaroxaban | 2 | 81 |
| apixaban | 6 | 650 | dabigatran | 7 | 1108 | dabigatran | 2 | 442 | prevention | 3 | 66 |
| dabigatran | 5 | 599 | apixaban | 7 | 812 | apixaban | 6 | 319 | enoxaparin | 3 | 56 |
| atrial fibrillation | 4 | 495 | atrial fibrillation | 5 | 677 | venous thromboembolism | 3 | 287 | deep-vein thrombosis | 2 | 45 |
| risk | 3 | 437 | management | 5 | 514 | atrial fibrillation | 2 | 247 | double-blind | 3 | 45 |
| management | 6 | 387 | stroke | 4 | 507 | prevention | 3 | 211 | thromboprophylaxis | 3 | 43 |
| stroke | 4 | 379 | venous thromboembolism | 2 | 503 | enoxaparin | 3 | 195 | bay-59-7939 | 1 | 40 |
| safety | 6 | 372 | risk | 4 | 501 | atrial-fibrillation | 5 | 171 | dabigatran etexilate | 4 | 36 |
| venous thromboembolism | 2 | 356 | safety | 5 | 501 | thromboprophylaxis | 3 | 167 | factor-xa inhibitor | 1 | 34 |
| direct oral anticoagulants | 1 | 329 | prevention | 2 | 407 | dabigatran etexilate | 3 | 161 | pharmacokinetics | 1 | 34 |
| anticoagulation | 2 | 312 | oral anticoagulants | 4 | 404 | stroke | 2 | 161 | bay 59-7939 | 1 | 29 |
| anticoagulants | 5 | 289 | atrial-fibrillation | 1 | 398 | anticoagulation | 2 | 154 | pharmacodynamics | 1 | 28 |
| atrial-fibrillation | 6 | 288 | anticoagulants | 4 | 392 | management | 2 | 154 | factor xa inhibitor | 2 | 26 |
| prevention | 2 | 278 | therapy | 4 | 387 | oral anticoagulants | 2 | 149 | total hip-replacement | 1 | 24 |
| oral anticoagulants | 4 | 240 | direct oral anticoagulants | 1 | 371 | safety | 1 | 146 | dabigatran | 2 | 22 |
| therapy | 5 | 210 | anticoagulation | 2 | 352 | anticoagulants | 1 | 145 | anticoagulants | 2 | 19 |
| thrombosis | 2 | 194 | efficacy | 5 | 299 | risk | 2 | 135 | direct thrombin inhibitor | 4 | 19 |
| efficacy | 4 | 193 | stroke prevention | 4 | 286 | pharmacokinetics | 1 | 134 | molecular-weight heparin | 3 | 19 |
| outcomes | 4 | 188 | metaanalysis | 6 | 265 | pharmacodynamics | 1 | 129 | safety | 1 | 16 |
| edoxaban | 1 | 184 | thrombosis | 2 | 238 | double-blind | 3 | 128 | warfarin | 2 | 16 |
| aspirin | 3 | 173 | edoxaban | 9 | 237 | factor-xa inhibitor | 1 | 128 | apixaban | 2 | 15 |
| pharmacokinetics | 1 | 169 | outcomes | 4 | 210 | therapy | 2 | 120 | total knee replacement | 1 | 15 |
| metaanalysis | 4 | 162 | bleeding | 9 | 195 | stroke prevention | 2 | 113 | antithrombotic agent | 1 | 14 |
| bleeding | 6 | 152 | aspirin | 3 | 186 | deep-vein thrombosis | 3 | 109 | fondaparinux | 2 | 14 |

Table S5 Top 10 most frequently cited articles.

| n | Title | DOI | Total number of citations | Number of citations per year |
|---|---|---|---|---|
| 1 | Rivaroxaban versus Warfarin in Nonvalvular Atrial Fibrillation | 10.1056/NEJMoa1009638 | 6300 | 450 |
| 2 | 2015 ESC Guidelines for the management of acute coronary syndromes in patients presenting without persistent ST-segment elevation Task Force for the Management of Acute Coronary Syndromes in Patients Presenting without Persistent ST-Segment Elevation of the European Society of Cardiology (ESC) | 10.1093/eurheartj/ehv320 | 3899 | 433.22 |
| 3 | Comparison of the efficacy and safety of new oral anticoagulants with warfarin in patients with atrial fibrillation: a meta-analysis of randomised trials | 10.1016/S0140-6736(13)62343-0 | 3564 | 324 |
| 4 | Antithrombotic Therapy for VTE Disease CHEST Guideline and Expert Panel Report | 10.1016/j.chest.2015.11.026 | 3409 | 378.78 |
| 5 | 2020 ESC Guidelines for the management of acute coronary syndromes in patients presenting without persistent ST-segment elevation | 10.1093/eurheartj/ehaa575 | 2403 | 600.75 |
| 6 | Oral Rivaroxaban for Symptomatic Venous Thromboembolism. | 10.1056/NEJMoa1007903 | 2392 | 159.47 |
| 7 | 2019 AHA/ACC/HRS Focused Update of the 2014 AHA/ACC/HRS Guideline for the Management of Patients With Atrial Fibrillation: A Report of the American College of Cardiology/American Heart Association Task Force on Clinical Practice Guidelines and the Heart Rhythm Society in Collaboration With the Society of Thoracic Surgeons | 10.1161/CIR.0000000000000665 | 2335 | 389.17 |
| 8 | 2019 AHA/ACC/HRS Focused Update of the 2014 AHA/ACC/HRS Guideline for the Management of Patients With Atrial Fibrillation A Report of the American College of Cardiology/American Heart Association Task Force on Clinical Practice Guidelines and the Heart Rhythm Society | 10.1016/j.jacc.2019.01.011 | 2335 | 389.17 |
| 9 | Antithrombotic Therapy for VTE Disease Antithrombotic Therapy and Prevention of Thrombosis, 9th ed: American College of Chest Physicians Evidence- Based Clinical Practice Guidelines | 10.1378/chest.11-2301 | 2228 | 171.38 |
| 10 | Oral Rivaroxaban for the Treatment of Symptomatic Pulmonary Embolism | 10.1056/NEJMoa1113572 | 1734 | 133.38 |

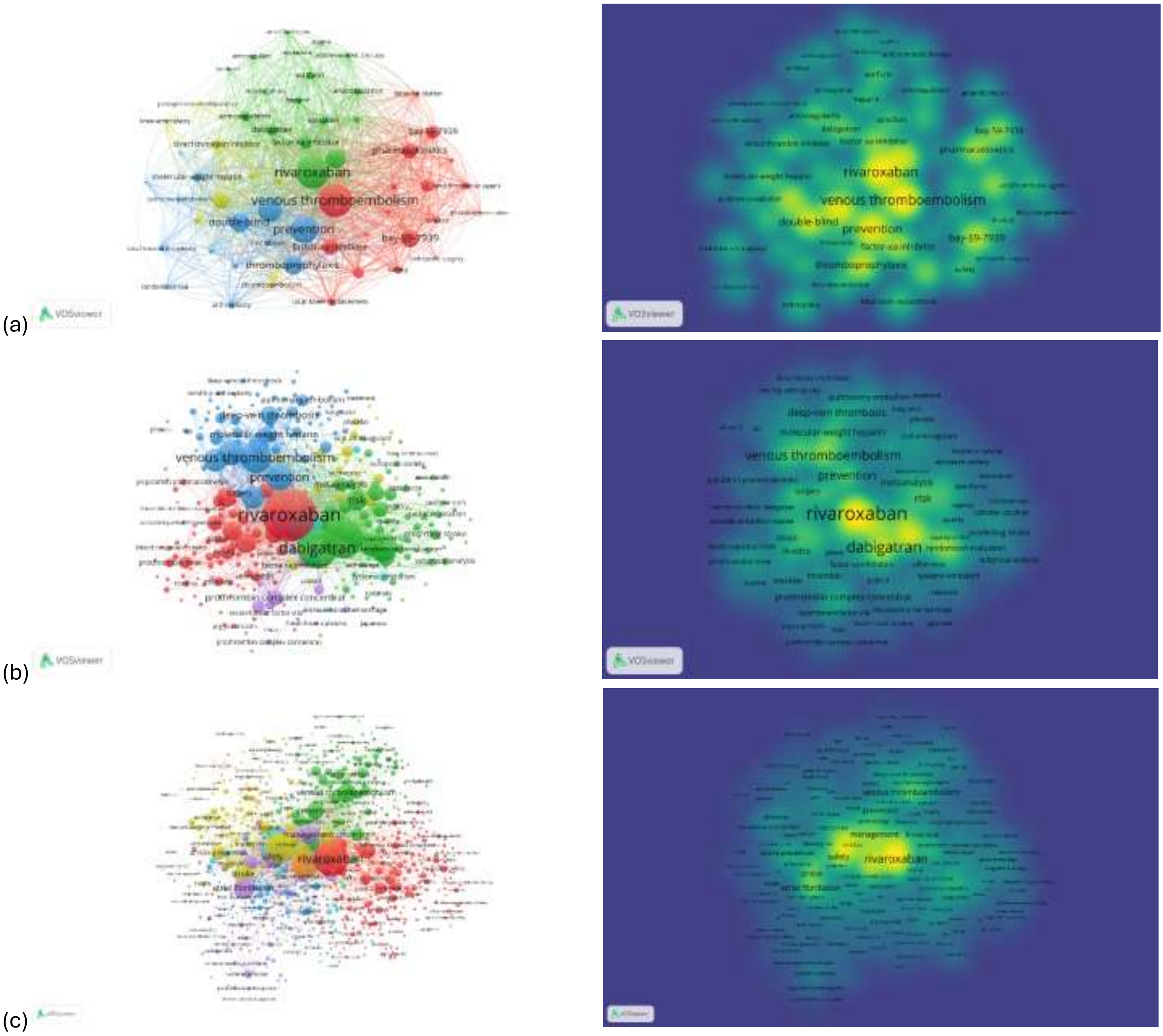


**Fig S1** Network map of rivaroxaban co-occurrence keywords including density distribution, between 2006-2010 (a), 2011-2015 (b) and 2016-2020 (c)
